# Bond strength of resin‐based restorative materials to fast‐setting calcium silicate cement using different resin adhesive systems

**DOI:** 10.1111/eos.13025

**Published:** 2024-10-27

**Authors:** Bahram Ranjkesh, Hilde M. Kopperud, Henrik Løvschall

**Affiliations:** ^1^ Section for Prosthetic Dentistry, Department of Dentistry and Oral Health Aarhus University Aarhus Denmark; ^2^ Nordic Institute of Dental Materials (NIOM) Oslo Norway; ^3^ Section for Oral Ecology, Department of Dentistry and Oral Health Aarhus University Aarhus Denmark

**Keywords:** adhesion, compomers, composite resins, dental adhesives, shear strength

## Abstract

This study assessed the bond strength of resin‐based restorative materials to fast‐setting calcium silicate cement (Aarhus Uinversity, Denmark) when treated with each of two one‐bottle universal adhesive systems. The cement surface (*N* = 256) was treated with a self‐priming adhesive and a self‐etch phosphate monomer‐containing adhesive with and without etching of the cement surface. Specimens then received either resin composite or compomer restorative materials (*n* = 32). The bond strength was measured after 1 day and 1500 thermocycles (*n* = 16). The failure type was visually inspected. The cement‐adhesive‐restorative material interface was visualized using scanning electron microscopy (SEM). The data were analyzed using multiple linear regression. Restorative material type, resin adhesive system, and thermocycling had a statistically significant effect on the bond strength. Compomer restorative material and self‐etch universal adhesive system demonstrated statistically significantly higher bond strength values to fast‐setting calcium silicate cement, predominantly exhibiting cement cohesive failure. Etching the cement surface enhanced the bond strength of the self‐priming universal adhesive. Thermocycling significantly reduced the bond strength. SEM showed self‐etch universal adhesive seemingly diffused over the etched cement surface compared to other groups. Self‐etch phosphate monomer‐containing universal adhesive and compomer resulted in the highest bond strength to fast‐setting calcium silicate cement.

## INTRODUCTION

The protection of the pulpo‐dentinal complex to preserve the vitality of the pulp organ is a crucial clinical concern in the operative treatment of tooth caries or traumatic dental injuries. Accordingly, it is important that dental materials, such as calcium hydroxide‐releasing calcium silicate cement, applied to the coronal portion of the tooth are biocompatible and can stimulate dentinogenesis and seal off against bacterial flux [1]. Reliable bonding between tooth structure and dental material will not only provide retention but also prevent bacterial leakage and enhance the outcomes of operative dental treatments [2].

Since the introduction of calcium silicate cements as a mineral trioxide aggregate (MTA) in the 1990s [3], calcium silicate cements have been used in a variety of endodontic treatments. Calcium silicate cements are hydrophilic and hard‐setting cements that form calcium silicate hydrate and calcium hydroxide as hydration by‐products after mixing with water [4]. The favorable biological properties of calcium silicate cement on pulp [5] and periapical tissues [6] have been attributed to the calcium hydroxide release [7]. Further, calcium hydroxide interacts with available environmental phosphate ions, leading to the formation of interfacial [8] and superficial apatite precipitates [9], both of which improve the sealing [10] and bonding of the cement to dentine [11]. Despite the advantages of MTA, its low initial mechanical strength [12], long setting time (that is, 2–3 h) [13, 14], poor handling characteristics [15], and risk of wash‐out upon contact with fluids [16] impede its practical use in the tooth crown.

With a view to support the application of calcium silicate cements in the crown, a fast‐setting calcium silicate cement has been developed at Aarhus University, Denmark. This white‐colored calcium silicate cement shares some components with MTA, including tricalcium silicate, dicalcium silicate, tricalcium aluminate, and calcium sulfate (calcium‐silicate‐aluminate composition: CaO 60%–70%, SiO_2_ 20%–30%, Al_2_O_3 _< 5%, tricalcium aluminate > 7%, lowered SO_4 _< 3%), while additional components include fluorides (3.5% wt.), phosphate, and zirconium oxide (10% wt.) as radio‐opaquer. This calcium silicate cement is a candidate for application as a cavity liner and for cementation in tooth crowns in addition to the traditional application of calcium silicate cements in the root canal. The practical consistency of the cement for the specific application is achievable with a slight adjustment of the powder‐to‐liquid ratio. As a result, the initial setting time of this calcium silicate cement ranges from 4 to 10 min depending on the cement's consistency [17].

The fast‐setting calcium silicate cement can form apatite precipitates over the cement's free surface [18] and within experimental interfacial gaps between cement and dentin submerged in phosphate‐buffered saline [19] due to calcium hydroxide release. The cytotoxicity of the fast‐setting calcium silicate cement is comparable to that of ProRoot MTA [20], with better mechanical properties (diametral tensile strength) than seen for ProRoot MTA and Biodentine [17]. Recent clinical studies have shown successful treatment outcomes with the fast‐setting calcium silicate cement in apexification of avulsed immature permanent incisors [21], direct pulp capping in primary dentition [22], and vital pulp therapy in permanent immature open‐apex teeth [23]. The fast‐setting calcium silicate cement outperformed the traditional glass ionomer and zinc phosphate cement in terms of bond strength when it was used for the cementation of intracanal titanium posts [24]. Further, the bond strength of the fast‐setting calcium silicate cement to dentin increases significantly over time in contrast to MTA (ProRoot MTA), glass ionomer cement (Ketac Molar), and calcium‐hydroxide lining material (Dycal) [25].

Contemporary resin adhesion strategies are catefgorized into etch‐and‐rinse and self‐etch, depending on how the resin adhesives interact with the smear layer formed during mechanical cavity preparation. Each adhesive strategy may include several steps depending on the product. These steps may include etching, priming, and bonding agent application separately, as done in the 3‐step adhesive technique or with etching and priming merged as 2‐step adhesive systems. Clinical interest in simpler and less technique‐sensitive systems has encouraged manufacturers to develop more user‐friendly adhesive systems [26]. One‐bottle dental adhesive systems, known as universal or multimode adhesives, provide the ability to apply the adhesive system either in etch‐and‐rinse, self‐etch, or an alternative “selective enamel etching” strategy [27]. The promising properties of the fast‐setting calcium silicate cement motivated the study of the interaction of this cement with different one‐bottle self‐priming and self‐etching universal adhesive systems and restorative materials, since the fast‐setting calcium silicate cement is a practical candidate for pulpal protection as a cavity liner before placement of the definitive restoration.

The aim of this study was to evaluate the bond strength of fast‐setting calcium silicate cement to resin‐based restorative materials after surface treatment using two one‐bottle universal adhesive systems with and without etching of the cement surface with phosphoric acid. The null hypotheses were that the bond strength of fast‐setting calcium silicate cement is not influenced by (i) resin‐based restorative material type, (ii) adhesive system, (iii) surface etching, and (iv) thermocycling.

## MATERIAL AND METHODS

The methodology for shear bond strength testing was adopted from ISO 29022:2013–Dentistry—Adhesion—Notched‐edge shear bond strength test [28]. Table 1 summarizes the materials used in the study. Specimen preparation and bond strength testing were performed in a climatized room with a controlled relative humidity of 50 ± 5 % and temperature of 23 ± 2°C.

**TABLE 1:** Composition of the tested materials.

Material	Composition
Fast‐setting calcium silicate cement	Powder: Tricalcium silicate, dicalcium silicate, tricalcium aluminate, calcium sulfate, fluoride additive (3.5%), radiocontrast (10%), nano‐SiO_2_ Liquid: 2% superplasticizer (polycarboxylic acid‐based) diluted in water
Prime & Bond XP Self‐priming universal adhesive for etch‐and‐rinse technique	Resin: UDMA ((di)urethane dimethacrylate), HEMA (2‐hydroxyethyl methacrylate), TEGDMA (triethyleneglycol dimethacrylate), PENTA (dipentaerythritol pentacrylate phosphate) phosphoric acid esters, TCB (Butan‐1,2,3,4‐tetracarboxylic acid, di‐2‐ hydroxyethylmethacrylate ester), Camphorquinone, Butylated benzenediol, tert‐Butanol Filler: Nanoscale functionalized filler
Prime & Bond active Self‐etch universal adhesive for etch‐and‐rinse and self‐etch techniques	Resin: Bisacrylamide‐based crosslinker, MDP (10‐methacryloyloxydecyl dihydrogen phosphate), PENTA (dipentaerythritol pentacrylate phosphate) phosphoric acid esters, isopropanol, and water
Ceram.x Universal Nano‐Ceramic Restorative	Resin: Methacrylate‑modified polysiloxane, dimethacrylate resin, fluorescent pigment, stabilizer, camphorquinone, ethyl 4‑(dimethylamino) benzoate, iron oxide pigments, aluminum sulfo‑silicate pigments Filler: Barium‑aluminum borosilicate glass (1.1–1.5 µm), methacrylate functionalized silicon dioxide nanofiller (10 nm)
Dyract eXtra, Universal Compomer Restorative	Resin: Urethane dimethacrylate (UDMA)‐Carboxylic acid modified dimethacrylate (TCB resin), Triethyleneglycol dimethacrylate (TEGDMA), Trimethacrylate resin, camphorquinone Filler: Strontium‐alumino‐sodium‐fluoro‐phosphor‐silicate glass, silicon dioxide, and Strontium fluoride
Acid etch agent	36% phosphoric acid

### Specimen preparation

Two‐hundred‐fifty‐six cylindrical epoxy molds, with a height of approximately 2 cm and a 3 cm diameter, were fabricated using a standard plastic mold. Briefly, two‐component embedding epoxy material (EpoFix Resin, Struers) was mixed according to the manufacturer's instruction, poured into the plastic molds, and kept inside a humid chamber overnight. Thereafter, the epoxy molds were removed from the plastic mold and the surface was plano‐parallelly polished (Planopol, Struers) with 500‐grit silicon carbide polishing paper (Struers). A central cavity with approximately 6 mm diameter × 4 mm height was created in the middle of the epoxy mold using a stationary drilling machine.

One gram of fast‐setting calcium silicate cement (Aarhus Uinversity, Denmark) was cap‐mixed (Silamat S6, Ivoclar Vivadent) with 180 µL hydration liquid containing 2% long‐chained polycarboxylic acid for 20 s. The resulting paste of fluid‐creamy consistency was filled into the cavity. The specimens were kept at room temperature within a humid chamber for 10 min to ensure the initial setting time of the cement before surface treatment.

### Surface treatment

Two universal one‐bottle adhesive systems, with and without surface etching with phosphoric acid, were used to treat the surface of the fast‐setting calcium silicate cement before placement of the restorative materials. The adhesive systems were self‐priming Prime & Bond XP (Dentsply DeTrey), which should be used with the etch‐and‐rinse technique, and self‐etch Prime & Bond active (Dentsply DeTrey), which can be used with both etch‐and‐rinse and self‐etch techniques. For both adhesives, we acid‐etched the fast‐setting calcium silicate cement surface for 10 s with 36% phosphoric acid (Acid etch, Conditioner 36, Dentsply DeTrey) followed by 5 s of rinsing with distilled water and 5 s drying with oil‐free airflow. After the application of the resin adhesive, the adhesive was light‐cured for 10 s using a dental light‐cure unit in homogenous irradiance mode (Demi Ultra, Kerr).

Accordingly, the fast‐setting calcium silicate cement surface was treated in one of the following four test groups (*n* = 64 per group):

- Group 1: etch, rinse, air‐dry, self‐etch universal adhesive, light cure.
- Group 2: rinse, air‐dry, self‐etch universal adhesive, light cure.
- Group 3: etch, rinse, air‐dry, self‐prime universal adhesive, light cure.
- Group 4: rinse, air‐dry, self‐prime universal adhesive, light cure.

Each group was subdivided by restorative material and aging protocol as explained below, resulting in 16 specimens per test group.

### Application of restorative materials

**FIGURE 1:**
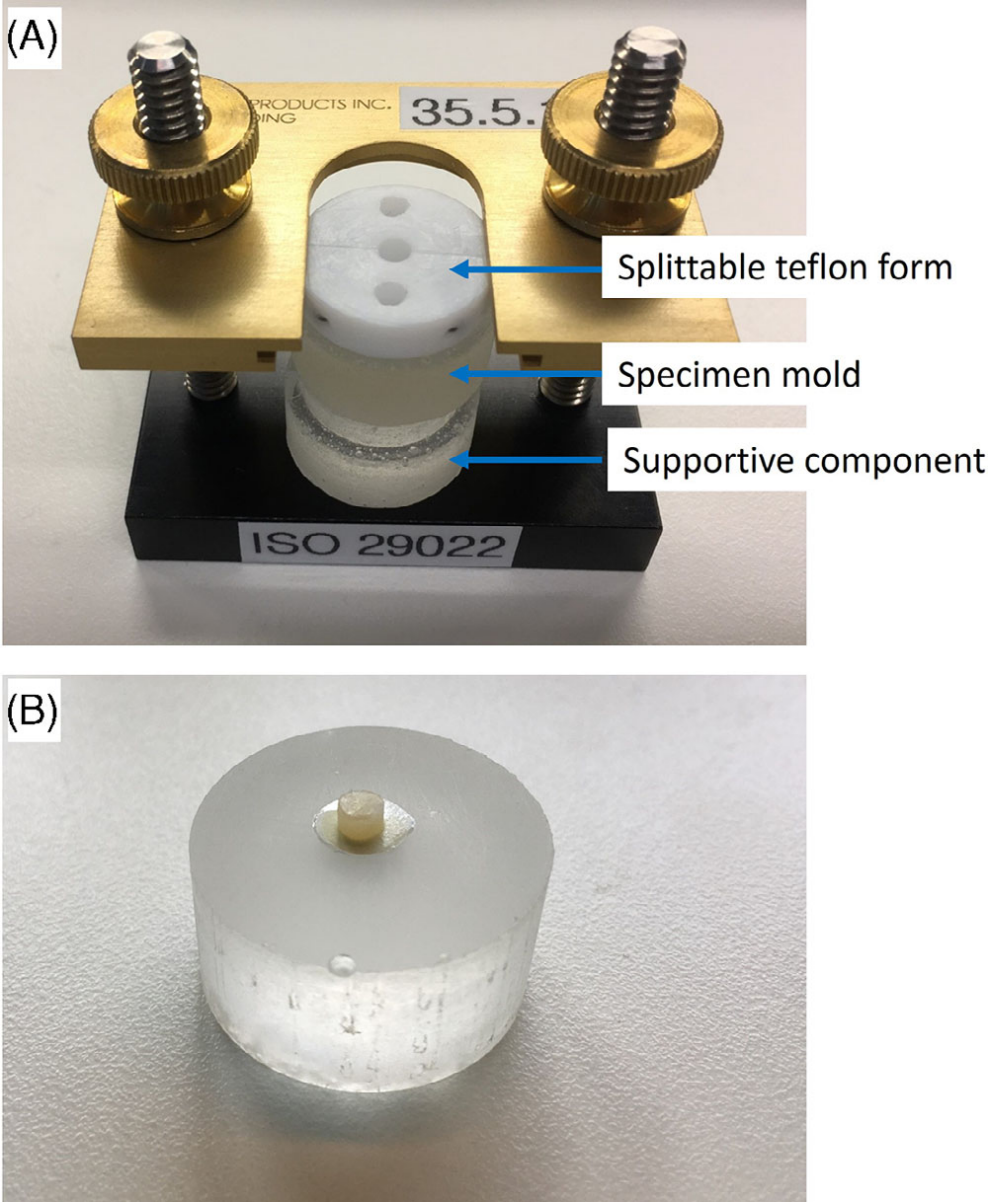
(A) The holder with different components according to ISO 29022 to make specimens for shear bond strength testing. (B) Specimen with restorative material.

Immediately after the curing of the adhesive, the restorative material was applied to the surface of the specimen. A splitable Teflon form with a central hole of 3 mm diameter was placed over the specimen and fixed onto the standard holder (Figure 1A). The resin‐based restorative materials used included a composite (Ceram.x Universal Nano‐Ceramic Restorative, Dentsply DeTrey) and a compomer (Dyract eXtra, Universal Compomer Restorative, Dentsply DeTrey), and each of these was filled into the hole of the mold (*n* = 32 per restorative material in each of groups 1 to 4). The restorative material was filled at a maximum height of 2 mm and light‐cured for 20 s using a dental light‐cure unit in homogenous irradiance mode (Demi Ultra, Kerr). Thereafter, the Teflon mold was gently split and withdrawn from the epoxy mold (Figure 1B).

### Shear bond strength test

Specimens from each of the eight experimental groups were then divided into two groups (*n* = 16 per subgroup), one in which bond strength was tested after 1 day of storage in a humid chamber at 37°C and one in which bond strength testing took place after 1500 thermocycles following 1 day of storage in a humid chamber at 37°C. Thermocycling was performed with 20 s of dwelling time in water baths with temperatures of 5°C and 55°C, respectively, and 5 s transfer time between baths. The shear bond strength testing was performed using a universal testing machine (Lloyd LRX: Lloyd Instruments) at the crosshead speed of 1.0 mm/min. The shear force was applied over the restorative material at the closest possible distance to the interface of the cement and restorative material. The highest load value before debonding was recorded in Newton. The bond strength in megapascal (MPa) was calculated by dividing the load value (Newton) by the bonded surface area (7.065 mm^2^). The mode of fracture after debonding was determined under a stereomicroscope at × 20 magnification as either adhesive (failure solely in the interface of the cement and restorative material), cohesive (failure within the cement or restorative material), or mixed failure (combination of adhesive and cohesive failure).

### Interface visualization using scanning electron microscopy (SEM)

Two specimens per combination of restorative material and surface treatment corresponding to groups 1 to 4 were fabricated for interface visualization. The specimens with the restorative material were fully embedded in epoxy material (EpoFix Resin, Struers) and sectioned perpendicular to the interface using a precision cutting machine (MICRACUT 201, Metkon) with a diamond saw under constant water cooling. Thereafter, the surface was polished with a sequence of silicon carbide polishing paper from grit 320 to 4000 (Struers) under water cooling. Gold/palladium sputtering coating was done (Sputter Coater SCD 050, Balzers) for 90 s. The interface visualization was performed using SEM (Philips XL, Philips) at an accelerating voltage of 16 kV.

### Statistical analysis

The data were analyzed using multiple linear regressions [proc regress of stata statistical software, release 12 (StataCorp)], with the type of restorative material (composite [ref]/compomer), adhesive system (Prime & Bond active [ref]/Prime & Bond XP), cement surface etching (no [ref]/yes), and time of assessment (1 day storage [ref]/thermocycling) as the main independent variables and their interaction as covariates. The significance level was set at 5%.

## RESULTS

Table 2 presents the mean values and standard deviations of the bond strength between the fast‐setting calcium silicate cement and the resin‐based restorative materials following various surface treatments of the cement and tested after 1‐day storage in humid conditions and after 1500 thermocycles. Table 3 summarizes the estimate of the differences in bond strength values attributable to each experimental factor in the form of regression coefficients for each main effect, and for possibly relevant interactions. The average bond strength value for the combination of reference groups, that is, composite with Prime & Bond active, no etching, and no thermocycling, amounted to 6.3 (95% CI = 5.7, 6.8) MPa (constant value in Table 3). The estimated main effects indicated that the compomer yielded an average bond strength that was 1.3 (95% CI = 0.8, 1.8) MPa higher than seen for the composite, while the Prime & Bond XP adhesive system resulted in an average bond strength that was 2.7 (95% CI = −3.2, −2.2) MPa lower than seen for the Prime & Bond active adhesive. Both these main effects (restorative and adhesive system) were statistically significant at *p* < 0.001. Thermocycling resulted in an average bond strength value that was 0.8 MPa lower than seen for the specimens tested after 1 day of storage under humid conditions (*p* < 0.05). Only one statistically significant interaction (*p* < 0.05) was observed, between the adhesive system used and the etching of the surface, indicating that etching with the Prime and Bond XP adhesive resulted in a bond strength that was an average of 1.1 MPa higher than that indicated by the sum of the main effects for this combination (−2.7 [Prime and Bond XP] + −0.2 [etching]). Apart from this, there was no statistically significant effect of surface etching (main effect = −0.2 MPa, 95% CI = −0.6, 0.3)

**TABLE 2:** The bond strength between fast‐setting calcium silicate cement and different resin‐based restorative materials after different surface treatments using adhesive systems with the frequency of failure types after various surface treatments on day 1 and after 1500 thermocycling.

Restorative material	Adhesive system	Cement's surface etching	Thermocycling	Bond strength (MPa)	Failure types % (cohesive/adhesive/mixed)
Composite	P&B active	No	No	6.5 ± 2.1	(94/0/6)
			Yes	5.6 ± 2.2	(81/0/19)
		Yes	No	5.9 ± 1.1	(88/12/0)
			Yes	5.2 ± 1.7	(56/38/6)
	P&B XP	No	No	3.7 ± 1.4	(38/12/50)
			Yes	2.2 ± 1.5	(19/81/0)
		Yes	No	3.6 ± 2.1	(25/19/56)
			Yes	3.0 ± 1.5	(19/63/18)
Compomer	P&B active	No	No	7.6 ± 2.2	(69/25/6)
			Yes	7.5 ± 1.8	(81/6/13)
		Yes	No	7.0 ± 2.5	(88/12/0)
			Yes	6.3 ± 2.1	(75/6/19)
	P&B XP	No	No	4.7 ± 1.8	(6/75/19)
			Yes	3.6 ± 2.1	(12/63/25)
		Yes	No	4.9 ± 2.7	(12/88/0)
			Yes	4.3 ± 2.2	(6/63/31)

*Note*: Values are presented in mean ± standard deviations in megapascal (MPa).

**TABLE 3:** Effect of main variables on bond strength value (MPa) to fast‐setting calcium silicate cement presented as regression coefficients estimating the difference between the actual category and the reference category.

	Regression coefficient β (95% CI)
**Restorative material**	
Composite	Ref.
Compomer	1.3 (0.8, 1.8)^**^
**Adhesive system**	
Self‐etch adhesive (Prime & Bond active)	Ref.
Self‐priming adhesive (Prime & Bond XP)	−2.7 (−3.2, −2.2)^**^
**Cement's surface etching**	
No etching	Ref.
Etching	−0.2 (−0.6, 0.3)
**Thermocycling**	
No	Ref.
1500 cycles	−0.8 (−1.2, −0.3)^*^
Constant (intercept)	6.3 (5.7, 6.8)
**Interactions**	
Restorative material × bonding system	−0.04 (−1.0, 0.9)
Bonding system × cement's surface etching	1.1 (0.2, 2.1)^*^
Restorative material × bonding system × thermocycling	−0.4 (−2.3, 1.6)
Restorative material × bonding system × cement's surface etching	0.5 (−1.4, 2.5)

*Note*: The 95% CI indicates the precision of the estimated difference.

* *p* < 0.05.

** *p* < 0.001.

**FIGURE 2:**
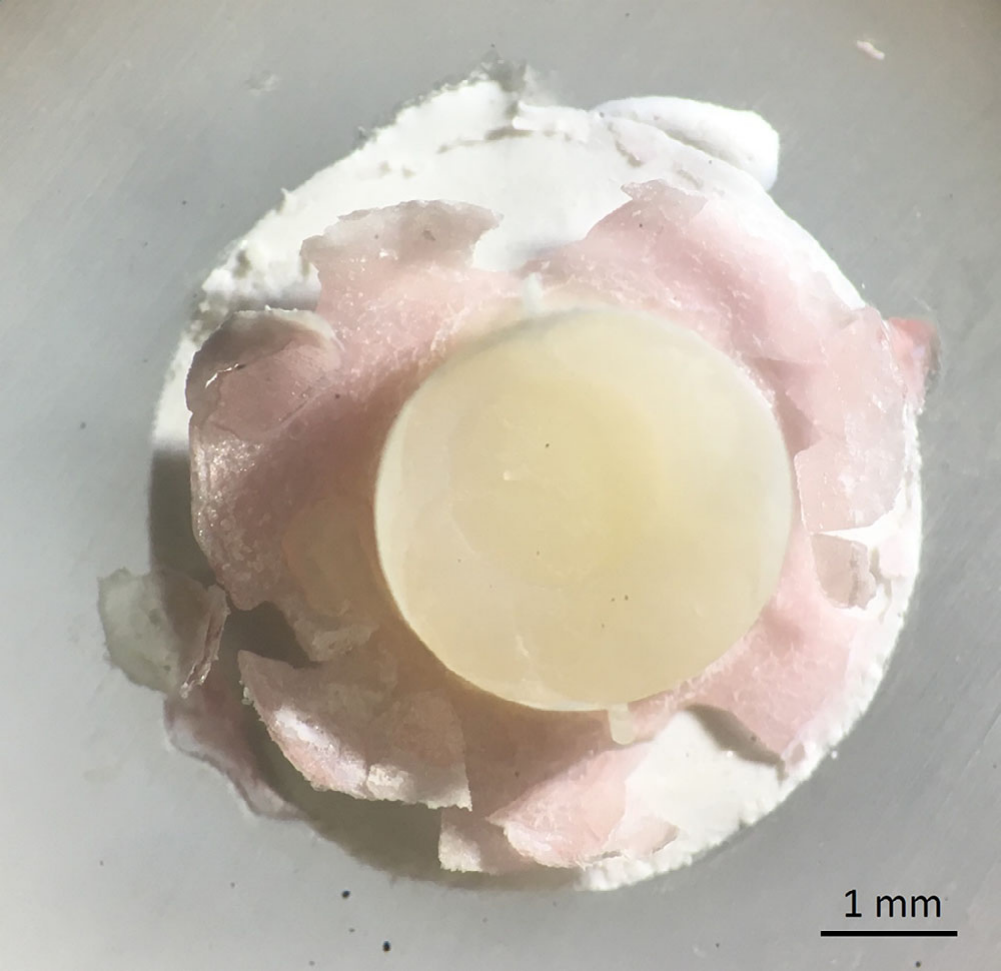
The representative sample under magnification visualizes the chemical interaction of the Prime & Bond XP adhesive system with the fast‐setting calcium silicate cement that forms a pink‐colored layer detached from the cement surface.

The cohesive failure within the cement was the most dominant failure type with the Prime & Bond active system, regardless of the restorative material type, cement surface etching, or thermocycling (Table 2). More adhesive failures were observed with Prime & Bond XP than with Prime & Bond active in the corresponding surface treatment groups. We also observed a few specimens with complete detachment of the restorative materials from the cement surface (pretesting failure). In the composite restorative material group, after cement surface etching and application of Prime & Bond XP, three and one samples were detached before and after thermocycling, respectively. Two composite and one compomer specimen detached in a solely applied Prime & Bond XP bonding system (without etching) after thermocycling. No pretesting failure was observed in groups with the Prime & Bond active universal adhesive system. The specimens with pretested failure received zero value in bond strength for statistical analysis. A chemical interaction was observed between the Prime & Bond XP adhesive system and the fast‐setting calcium silicate cement in the form of a pink‐colored superficial layer detaching from the cement's free surface after 1 day of storage of the specimen in humid conditions (Figure 2).

**FIGURE 3:**
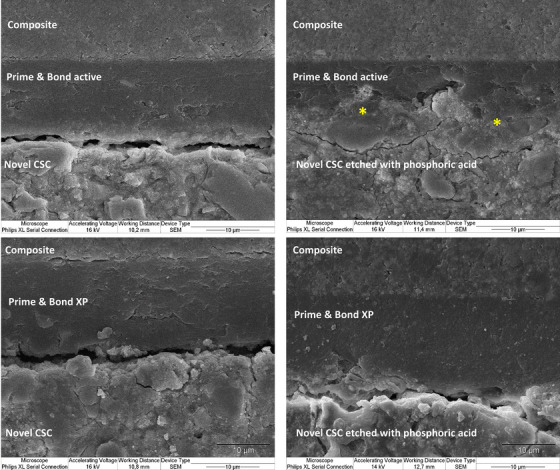
The visualization of composite restorative material and the fast‐setting calcium silicate cement (labeled novel CSC) interface after application of different adhesive systems. * suggests a possible diffusion of the Prime & Bond active adhesive system at the fast‐setting calcium silicate cement interface over the etched cement surface.

**FIGURE 4:**
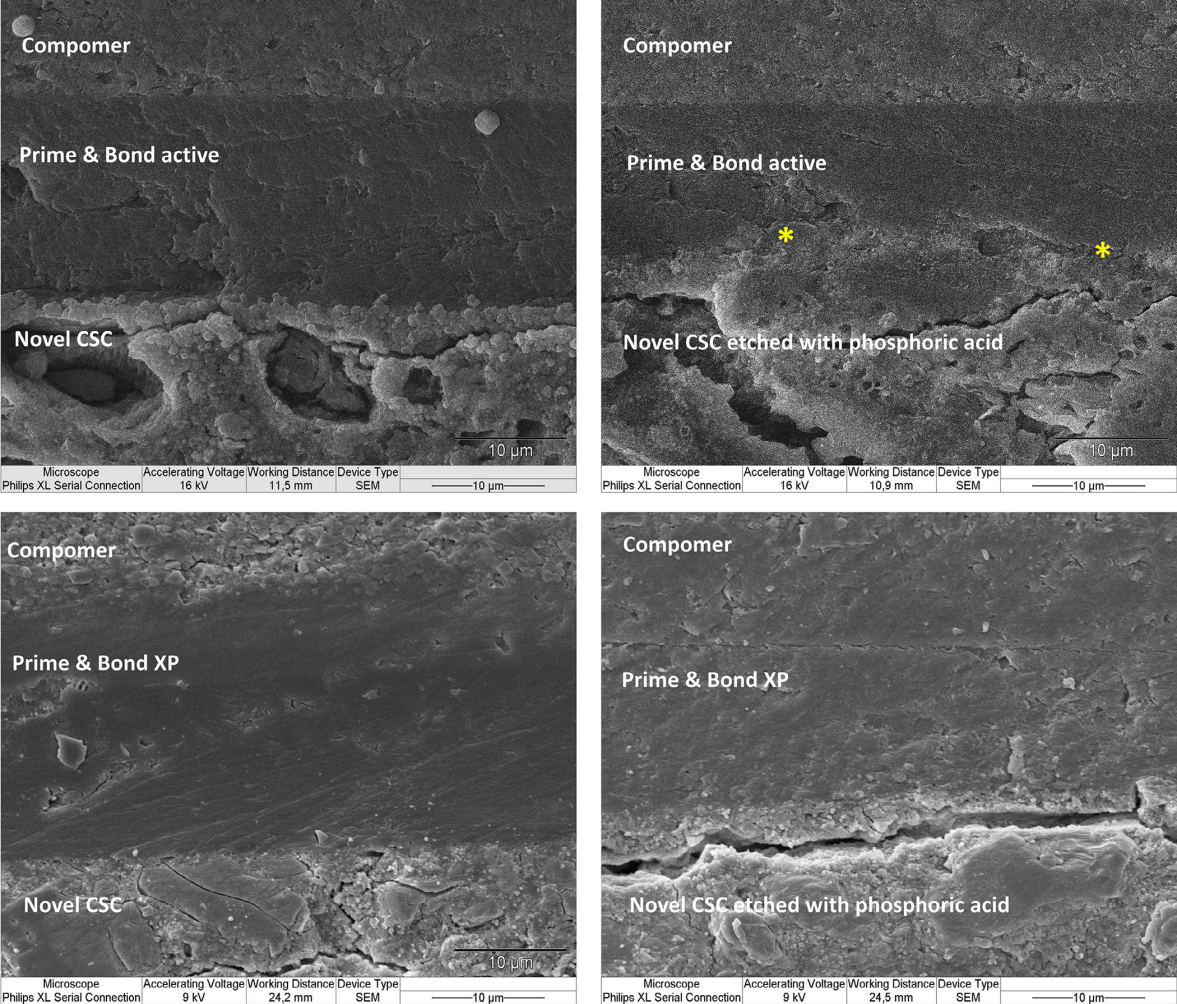
The visualization of compomer restorative material and the fast‐setting calcium silicate cement (labeled novel CSC) interface after application of different adhesive systems. * suggests a possible diffusion of the Prime & Bond active adhesive system at the fast‐setting calcium silicate cement interface over the etched cement surface.

**FIGURE 5:**
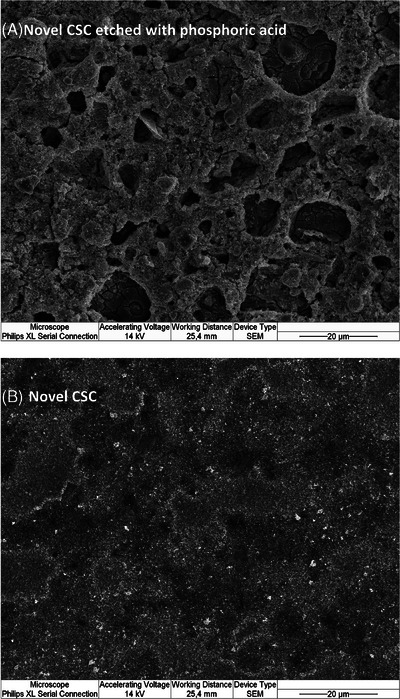
Topographic changes in terms of particle depletion and porous surface formation over the fast‐setting calcium silicate cement (labeled novel CSC) were observed (A) after 10 s of etching with 36% phosphoric acid compared to (B) the non‐etched cement surface.

Interface visualization with SEM revealed three distinguishable layers identified as restorative material, adhesive system, and fast‐setting calcium silicate cement (Figures 3 and 4). We observed that the interface between Prime & Bond active and etched fast‐setting calcium silicate cement was different from that seen in the other groups. Seemingly, the Prime & Bond active universal adhesive system diffused over the etched cement surface at the interface (Figures 3 and 4). This observation was evident with both types of restorative materials. Figure 5 represents the observable topographic alterations over etched fast‐setting calcium silicate cement in the form of cement particle dissolution and opening of porous structures (Figure 5A) compared to the non‐etched cement surface (Figure 5B).

## DISCUSSION

This study assessed the bond strength of the fast‐setting calcium silicate cement when used in combination with different resin‐based restorative materials and after different cement surface treatments with different universal adhesive systems before and after thermocycling. The results showed that restorative material type, resin adhesive system, surface etching, and thermocycling influenced the bond strength of the fast‐setting calcium silicate cement. Therefore, the null hypotheses were rejected. Calcium‐hydroxide‐based materials traditionally are the clinical standard for cavity lining. The bond strength of calcium‐hydroxide‐based materials (Dycal, Dentsply) has been reported in the mean range of 0.9 MPa to 2.2 MPa to resin composite depending on the adhesive system [29]. Despite the methodological variation, the obtained data in our study for bond strength of fast‐setting calcium silicate cement to resin restorative materials using the applied adhesive systems may seem clinically sufficient. This study demonstrated the highest bond strength to the fast‐setting calcium silicate cement of the compomer restorative material and self‐etch one‐bottle universal adhesive.

Macro‐shear bond testing is one the most commonly used methods to evaluate the bond strength between different substrates [30]. The method is popular, rather easy, and fast. However, different factors, such as the e‐modulus of the material, aging conditions (e.g., thermocycling), and test design (e.g., crosshead speed, configurations of shear force applier), are among the limiting factors that may influence the results [31]. The uniformity of stress distribution at the true interface has also been questioned for the planar shear bond test [32]. Despite the limitations of the method, it is believed that the shear bond strength test remains a practical test to evaluate bonding effectiveness [30].

Reliable enamel bonding is predictably achievable by the application of phosphoric acid where the interlocking of resin tags is formed over etched enamel [33]. However, durable resin bonding to dentin has always been a challenge because of the significant amount of water and organic collagenous material in the substrate [34]. This has resulted in different adhesive systems, such as 3‐step etch‐and‐rinse adhesives, 2‐step etch‐and‐rinse adhesives, 2‐step self‐etch adhesives, and simple one‐step universal adhesives, currently being available to counter the problem. Although conventional 3‐step etch‐and‐rinse adhesives and (mild) 2‐step self‐etch adhesives are still the points of reference for dental adhesion in routine clinical practice [30], the simplicity of the one‐step universal adhesives makes them attractive for clinicians.

In this study, we tested self‐priming Prime & Bond XP, which is indicated for etch‐and‐rinse techniques, and self‐etch Prime & Bond active, which can be applied both in etch‐and‐rinse and the self‐etch techniques according to the manufacturer's instruction. Our results showed that Prime & Bond active resulted in significantly higher bond strength to the fast‐setting calcium silicate cement than the Prime & Bond XP bonding system. The chemical composition of each adhesive system may explain its interaction and bonding with the calcium silicate cement surface. Prime & Bond XP is a conventional “acryl” resin mixture with adjusted viscosity comprising UDMA ([di]urethane dimethacrylate), HEMA (2‐hydroxyethyl methacrylate), TEGDMA (triethyleneglycol dimethacrylate), PENTA (dipentaerythritol pentacrylate phosphate) phosphoric acid esters, and butanol, according to the manufacturer's information. Prime & Bond active is a hydrophilic and mild‐etch (pH value > 2.5) adhesive formulation comprising polymerizable bisacrylamide‐based crosslinker, 10‐MDP (10‐methacryloyloxydecyl dihydrogen phosphate), and PENTA phosphoric acid esters, isopropanol, and water, according to the manufacturer's information. The presence of water as the solvent in the composition of Prime & Bond active may result in this adhesive system being more compatible with hydrophilic calcium silicate cement than Prime & Bond XP, where the composition reveals an adhesive of intermediate hydrophilicity. Furthermore, the Prime & Bond active contains an acidic 10‐MDP functional phosphate monomer, which is often used in adhesive technology because it can bond to both inorganic and organic structures. The 10‐MDP has a low pH and will etch the dentin, leading to the release of calcium ions, whereby both the formation of stable 10‐MDP‐calcium salts [35] and ion bonding to hydroxyapatite may be supported. This may provide an acid‐base stable chemical adhesion to the tooth structure, when compared to micromechanical adhesion only [35, 36]. We do not yet fully understand the complete bonding mechanism of 10‐MDP to the fast‐setting calcium silicate cement, but we speculate that, although the fast‐setting calcium silicate cement releases calcium hydroxide (which can be expected to neutralize the adhesive's low pH), the presence of a substantial amount of calcium ions may contribute to the improved bonding ability of 10‐MDP‐containing adhesive system to calcium silicate cement. Meraji and Camilleri [59] suggested that the use of a self‐etch adhesive system (without 10‐MDP monomer) over calcium silicate cement (Biodentine) results in poor bonding and even complete pretest debonding of the composite resin restorative material during thermocycling. In contrast, the use of 10‐MDP containing self‐etch bonding systems showed increased bond strength of calcium silicate cement to resin composite restorative materials [37].

Etching with 36% phosphoric acid only had a statistically significant effect on the bond strength of the fast‐setting calcium silicate cement when the self‐priming Prime & Bond XP adhesive was used. The observation of pretest adhesive failures in the Prime & Bond XP group and the observation of problematic interfacial interaction with an exfoliating pink‐colored layer on the cement surface (Figure 2) indicated that the Prime & Bond XP bonding to the fast‐setting calcium silicate cement is inadequate and presumably achievable with only micromechanical retention. The detachment of the Prime & Bond from the calcium silicate cement surface requires further study. However, the observation of a higher bond strength when the Prime & Bond active was used on the fast‐setting calcium silicate cement and the observation that the bond strength exceeded the cement's mechanical properties, leading to dominance of cohesive failures within the fast‐setting calcium silicate cement, suggests that the adhesive‐calcium silicate cement interaction is more adequate. It is important to recognize that the setting reaction and hydration of calcium silicate cement require time, potentially extending over several weeks, in which time the mechanical properties gradually improve [38]. In this study, bond strength was evaluated after 1 day, a period during which the cement is not fully set and its mechanical properties have not reached their maximum values [17]. This factor may contribute to the observation of more cohesive failures within the cement and may thus influence the results. Further studies to assess the effect of time on bond strength seem relevant. In SEM observations, the Prime & Bond active system exhibited a tendency to diffuse into the etched cement surface (Figure 3 and Figure 4). This fact may indirectly suggest the hydrophilicity of this adhesive system enabling it to incorporate with the hydrophilic cement structure. However, further chemical analysis seems essential to elaborate on this interaction.

Our findings demonstrated that compomer restorative material had significantly higher bond strength to the fast‐setting calcium silicate cement than resin composite restorative material both before and after thermocycling. Compomers or polyacid‐modified resin composites were introduced as an aesthetic posterior and anterior restorative material with fluoride release in 1990s [39] with successful 3‐year clinical outcomes [40], mainly being used in primary dentition [41]. However, the clinical success rates of using compomer without adhesive are poor, and it is generally recommended to use one‐step adhesive systems with compomer [42]. Similar to resin composites, compomers are fundamentally considered hydrophobic, but they contain hydrophilic components as acid‐functional monomers [43], which make compomers more hydrophilic than resin composites [44]. Therefore, the relative hydrophilicity of the compomer may be a contributing factor that explains the better bond strength of the compomer to the fast‐setting calcium silicate cement.

In contrast to our findings, other studies have shown that either resin composite or compomer displayed no statistically significant differences in their bond strength to MTA [45, 46]. The application of a bonding agent over even freshly mixed MTA is essential to obtain a gap‐free interface with resin composite restorative material [47]. Although calcium silicate cements contain large amounts of calcium oxide, methodological variations, including differences in the setting kinetics, setting time, and mechanical strength development of calcium silicate cements, may influence the results. The elapsed time allowing the setting of the calcium silicate cements and the use of different bonding systems may explain the discrepancies. Besides the difference in hydrophilicity of compomer and composite, the modulus of elasticity, in general, influences the findings of the shear bond test: the higher the modulus of elasticity, the higher the bond strength value [31]. However, Sochacki et al. [48] showed that the flexure modulus of composite (Ceram.x Universal) and compomer (Dyract eXtra) did not differ statistically significantly, whether after short‐ (1 h) or long‐term (1 month) storage in physiologic phosphate‐buffered saline solution [48].

Thermocycling is an artificial in vitro aging method to test bond durability, where the specimens are subjected to temperature changes similar to those experienced in vivo [49]. Thermal stresses challenge the bond between the adhesive and the tooth and, depending on the adhesive system, this may affect bond strength [50]. The temperature gradients and possible absorption of water by the dental materials induce alterations in material properties during thermocycling, which will eventually weaken the bond strength between the substrates by repetition of the thermal cycles [51]. The thermocycling process can adversely affect the interfacial bonding process and, eventually, the bond strength of calcium silicate cements to dentine [52]. Furthermore, mechanical stresses caused by differences in the coefficient of thermal expansion can additionally challenge the bonding interface [53]. It has been estimated that approximately 10,000 cycles represent 1 year of clinical service in vivo [49]. In the present study, 1500 cycles were used in a thermocycling process, which therefore resembles a period of approximately 2 months of in vivo service. This thermocycling process significantly decreased the bond strength between the fast‐setting calcium silicate cement and the resin‐based restorative materials. Short thermocycling periods have been shown to be sufficient to demonstrate a decrease in the bond strength of resin‐based composite‐to‐composite bonding [51, 54].

Implementation of thermocycling in the assessment of the bond strength of different restorative materials to different calcium silicate cements has not often been evaluated in earlier studies [55, 56, 57, 58]. One study showed an extremely weak bond with complete failure of the bond between the restorative material and calcium silicate cement (Biodentine, Septodont) after thermocycling [59]. Therefore, it has been suggested to delay the placement of overlying resin composite restoration over Biodentine for more than 2 weeks to allow sufficient intrinsic long‐term setting and maturation of the cement [56]. In contrast, another study showed that the bond strength of Biodentine and MTA (MTA Angelus, Angelus) to resin‐based flowable lining materials, when using a self‐etch adhesive, was comparable whether applied at 3 min, 15 min, or 2 days after mixing [60]. A wide range of variations makes it difficult to compare the studies. We applied the adhesive and restorative materials over the fast‐setting calcium silicate cement only 10 min after mixing, mimicking a one‐visit dental restorative treatment session. Interestingly, the highest bond strength values of compomer and self‐etch universal adhesive to fast‐setting calcium silicate cement were not influenced significantly by the thermocycling. The higher bonding of specimens with compomer and self‐etch universal adhesive to the fast‐setting calcium silicate cement seemed unaffected by the comparison of bonding before (7.6 ± 2.2 MPa) and after thermocycling (7.5 ± 1.8 MPa). Further studies comparing the different calcium silicate cements are needed.

Achieving a completely gap‐free composite‐to‐dentine bonding has been questioned, even with contemporary adhesive dentine systems [61, 62]. Therefore, the application of fast‐setting calcium silicate cement beneath the overlaying restorative material may clinically provide a biocompatible material [20] to maintain the pulpal vitality [22, 23] with simultaneous potential for closure of interfacial gaps between the cement and tooth substance [18]. Within the limitations of the current study, the application of a self‐etch universal adhesive system containing a phosphate‐modified monomer and a compomer restorative material resulted in the highest bond strength to fast‐setting calcium silicate cement.

## AUTHOR CONTRIBUTIONS

**Conceptualization**: Bahram Ranjkesh; Hilde M. Kopperud and Henrik Løvschall. **Methodology**: Bahram Ranjkesh and Hilde M. Kopperud. **Validation**: Bahram Ranjkesh and Hilde M. Kopperud. **Investigation**: Bahram Ranjkesh; Hilde M. Kopperud and Henrik Løvschall. **Formal analysis**: Bahram Ranjkesh. **Data curation**: Bahram Ranjkesh and Hilde M. Kopperud. **Writing—original draft preparation**: Bahram Ranjkesh. **Writing—review and editing**: Bahram Ranjkesh; Hilde M. Kopperud and Henrik Løvschall. **Resources**: Henrik Løvschall. **Visualization**: Bahram Ranjkesh; Hilde M. Kopperud and Henrik Løvschall. **Project administration**: Bahram Ranjkesh and Hilde M. Kopperud. **Funding acquisition**: Bahram Ranjkesh; Hilde M. Kopperud and Henrik Løvschall. **Supervision**: Hilde M. Kopperud.

## CONFLICT OF INTEREST STATEMENT

Henrik Løvschall has a conflict of interest as the patentee. Bahram Ranjkesh and Hilde M. Kopperud declare no conflicts of interest.

## Data Availability

Data are available on request from the authors.
